# Reply to Ott, M.; Werneke, U. Comment on “Liu et al. Hemodialysis Treatment for Patients with Lithium Poisoning. *Int. J. Environ. Res. Public Health* 2022, *19*, 10044”

**DOI:** 10.3390/ijerph20115951

**Published:** 2023-05-25

**Authors:** Yu-Hsin Liu, Tzung-Hai Yen

**Affiliations:** 1 Department of Anaesthesiology, Chang Gung Memorial Hospital, Linkou Branch, Taoyuan 333, Taiwan; mpq192@cgmh.org.tw; 2 Department of Nephrology, Clinical Poison Center, Chang Gung Memorial Hospital, Linkou Branch, Taoyuan 333, Taiwan; 3 College of Medicine, Chang Gung University, Taoyuan 333, Taiwan

We would like to thank Professor Ott and Professor Werneke for their helpful comments [1]. Importantly, the mortality rate related to lithium poisoning is zero in the study by Ott et al. [2]. Furthermore, lithium is freely filtered in the glomerulus and largely reabsorbed in the proximal tubule [3].

We agree that hemodialysis is an important procedure for treating patients with severe lithium poisoning as it increases lithium clearance. Nevertheless, the published hemodialysis rates for lithium poisoning vary widely, from 1.5% to 60.8% [4]. This is because there are some risks associated with emergency hemodialysis. Another explanation is that emergency hemodialysis intervention accessibility varies across different hospitals. Complications during femoral vein catheterization in emergency hemodialysis comprise infection, thrombosis, arterial puncture, groin hematoma, etc. [5]. Furthermore, complications of hemodialysis include heart arrhythmias, intradialytic hypotension or hypertension, dialysis disequilibrium syndrome, seizure, etc. Interestingly, Buckley et al. [6] commented that the hemodialysis criteria of the EXtracorporeal TReatments In Poisoning (EXTRIP) workgroup [7] were too broad, exposing lithium patients who may have recovered with conservative treatment to the risks of hemodialysis and incurring additional costs. Through an integrated assessment of the pattern of poisoning, serum lithium level, renal function and clinical presentation of toxicity, they were able to reduce the need of hemodialysis but still identify the patients who benefit from it. In summary, our group advocates that hemodialysis treatment for lithium poisoning should be patient-tailored, and there is no ideal universal indication for all lithium patients.
